# Management of a Failed Distal Tibial Allograft Procedure for Anterior Shoulder Instability in a Patient With Epilepsy: A Case Report

**DOI:** 10.7759/cureus.51477

**Published:** 2024-01-01

**Authors:** John Grossi, Paul Danahy, Oren D Rosenthal, George P Ackerman

**Affiliations:** 1 Osteopathic Medicine, Lake Erie College of Osteopathic Medicine, Bradenton, USA; 2 Orthopaedic Surgery, Lake Erie College of Osteopathic Medicine, Bradenton, USA; 3 Anatomy, Lake Erie College of Osteopathic Medicine, Bradenton, USA; 4 Orthopaedic Surgery, Optum Urgent Care - Glen Oaks, Lake Success, USA

**Keywords:** autograft, allograft, latarjet, epilepsy, shoulder dislocation

## Abstract

Shoulder instability episodes are observed in high-energy injuries, such as seizures. In this case report, we highlight the management of a failed distal tibial allograft procedure for recurrent shoulder instability in a patient with a bony Bankart lesion and epilepsy. The patient was treated with an iliac crest autograft and a proximal humerus osteochondral allograft procedure. To our knowledge, the use of an iliac crest autograft for glenoid bone loss and a proximal humerus osteochondral allograft after several failed shoulder instability procedures in a patient with epilepsy has not been reported.

## Introduction

Shoulder instability is common in patients with seizure disorders [1]. Although conservative treatment for shoulder instability is often implemented, the risk of recurrent instability in patients younger than 20 years is significant [2]. High-energy mechanisms of injury and recurrent shoulder instability can result in a fracture of the anterior-inferior aspect of the glenoid, referred to as a bony Bankart lesion [1]. The mechanism of shoulder dislocation can lead to a lesion of the humeral head, known as a Hill-Sachs lesion [2]. Remplissage is a technique in which large Hill-Sachs lesions are filled to smoothen the posterior humerus [2]. Several surgical procedures exist to treat bony Bankart lesions, such as open Latarjet, arthroscopic Latarjet, and the Bristow procedure [3]. The Latarjet procedure involves cutting the coracoid process and transferring it to the inferior glenoid with the conjoint tendon attached [3]. The Bristow procedure is similar to the Latarjet procedure but differs by using the osteotomy face of the coracoid process and fixating it to the glenoid rather than the horizontal surface of the coracoid graft [3]. This Bristow procedure was not indicated at this time because it is a relatively old technique with more complications compared to the Latarjet procedure [3]. The specific procedure indicated is based on the extent of glenoid bone loss [3]. Patients with more than 25% of glenoid bone loss are often recommended for the Latarjet procedure [3]. The average risk of recurrent instability following arthroscopic Bankart repair is 9%, with a range of 0% to 30% [4]. The risk of recurrent instability following an open Latarjet procedure ranges from 2% to 14% [4]. Several graft options can be utilized to provide further stability to the glenohumeral joint and aid the healing of the glenoid bone.

## Case presentation

**Figure 1:**
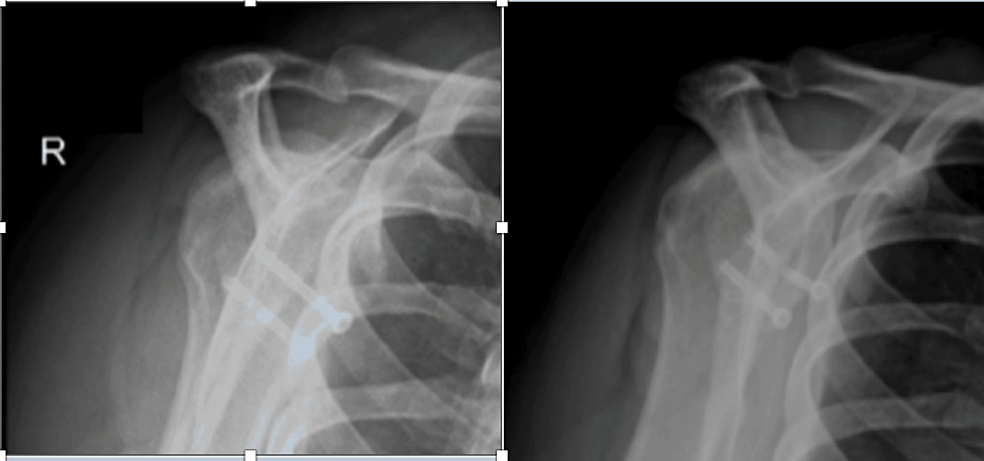
Scapular Y radiographic view at 1 week (left) and 4.5 months (right) after the distal tibial allograft procedure.

**Figure 2:**
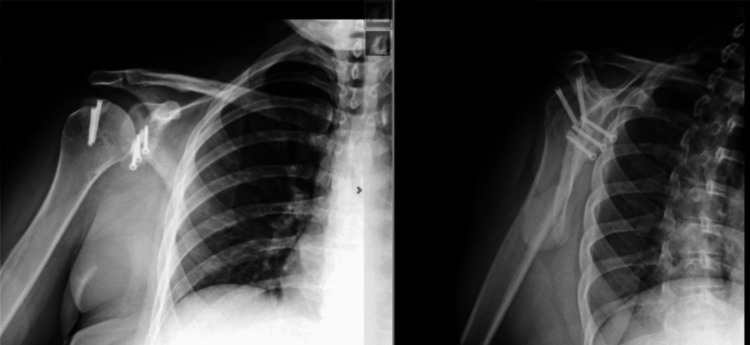
Anteroposterior (AP) and scapular Y radiographic views at 2 weeks after right shoulder iliac crest autograft glenoid fixation with proximal humerus osteochondral bone wedge allograft procedure.

**Figure 3:**
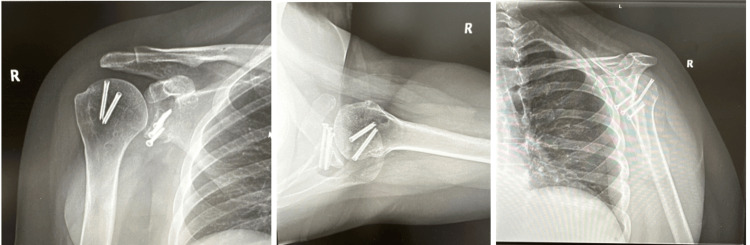
Anteroposterior (AP), axillary, and scapular Y radiographic views (left to right) at 12 months after right shoulder iliac crest autograft glenoid fixation with proximal humerus osteochondral bone wedge allograft procedure.

A 31-year-old female patient with a history of idiopathic generalized epilepsy was initially treated with arthroscopic Bankart repair several years prior to our initial consultation. Her epilepsy has been managed with lamotrigine 150mg and clobazam 20 mg, along with a vagal nerve stimulator. Due to persistent tonic-clonic seizures during the early postoperative period, she sustained a dislocation. The patient subsequently underwent a revision arthroscopic Bankart repair. Approximately five months following her revision Bankart repair, she sustained a shoulder dislocation while sleeping. A follow-up CT arthrogram revealed significant bone loss in the anterior-inferior glenoid. A procedure using a fresh distal tibial allograft was performed to further stabilize the shoulder and to avoid potential stress on a standard Latarjet bone graft from the coracoid connection to the conjoined tendon, given her history of epileptic seizures. Intraoperatively, the shoulder was taken through its range of motion after the graft placement without engagement of the Hill-Sachs lesion on the distal tibial allograft; therefore, no remplissage was indicated at this time. Approximately four and a half months following the distal tibial allograft procedure, the patient stated that her shoulder dislocated during a seizure. Radiographs performed in the office following this episode revealed that she fractured the cannulated screws and displaced the distal tibial allograft, as shown in Figure 1 in comparison with the postoperative radiographs one week after the distal tibial allograft procedure. The patient was advised to proceed with the revision procedure for stabilization purposes using an iliac crest bone autograft. The surgeon chose the iliac crest autograft for this revision procedure rather than the distal tibial allograft to further optimize the healing of the graft to the glenoid. In addition to the glenoid grafting, the Hill-Sachs deformity was addressed using a fresh proximal humerus osteochondral bone wedge allograft. A subscapularis tenotomy and capsular repair were performed for both the distal tibial allograft procedure and the iliac crest autograft procedure. Postoperative radiographs taken at the end of the 2-week and 12-month periods are shown in Figure 2 and Figure 3, respectively. Excellent graft integration was noted, and the patient’s shoulder has remained stable despite several subsequent seizures. Her vagal nerve stimulator has been adjusted by her neurologist throughout these procedures.

## Discussion

This case presentation highlights an alternative technique for addressing recurrent shoulder instability with associated bone loss in a patient with epilepsy. In this case, the iliac crest autograft procedure ultimately provided more rapid and reliable healing and long-term stability compared to the initial distal tibial allograft procedure.

In patients with seizure disorders, there is no gold standard procedure for shoulder instability. Patients with a seizure disorder have an increased chance of experiencing a seizure during the postoperative period, which in turn increases their risk of recurrent instability [1,5]. Therefore, controlling seizures during the preoperative and postoperative periods is recommended to prevent the recurrence of shoulder dislocation.

With recurrent shoulder instability, particular attention must be paid to glenoid bone loss and Hill-Sachs lesions [6]. In a standard Latarjet procedure, a coracoid autograft is used to augment the glenoid fossa. Other graft options include iliac crest autograft or distal tibial allograft [7,8]. We were unable to identify any studies comparing the efficacy of iliac crest autograft and distal tibial allograft procedures for glenoid bone loss in shoulder instability. The distal tibial allograft provides a true osteochondral allograft with a large bone stock and a similar radius of curvature to the native glenoid, along with a robust articular chondral surface [9]. Meanwhile, the iliac crest autograft has shown improved congruity of the glenoid as well as limitless bone graft size [10]. The standard Latarjet procedure using a coracoid autograft in patients with epilepsy could lead to excessive forces on the hardware due to muscle contractions that pull on the conjoint tendon, potentially causing hardware failure and recurrent instability [11]. A noted disadvantage with allografts is the potential for a lack of graft incorporation, while one of the main disadvantages of using an autograft is donor site morbidity and an increased risk of infection from the harvest site [12].

Many reports have indicated the use of a distal tibial allograft bone block procedure or an iliac crest bone block. However, there has not been conclusive data on which procedure provides superior outcomes, especially in patients with epilepsy. A standard Latarjet procedure was avoided due to her seizure disorder and concern that muscle contractions may pull on the coracoid graft.

This case report demonstrates that the iliac crest autograft provided better graft incorporation compared to the distal tibial allograft at a similar time interval during the postoperative period.

## Conclusions

The use of an iliac crest autograft for glenoid bone loss and recurrent instability is a viable alternative treatment option, particularly useful in treating patients with epilepsy as it avoids excessive stress on the coracoid bone graft of a standard Latarjet procedure during a seizure episode.
